# Effects of Rhythmic Transcranial Magnetic Stimulation in the Alpha-Band on Visual Perception Depend on Deviation From Alpha-Peak Frequency: Faster Relative Transcranial Magnetic Stimulation Alpha-Pace Improves Performance

**DOI:** 10.3389/fnins.2022.886342

**Published:** 2022-06-17

**Authors:** Andra Coldea, Domenica Veniero, Stephanie Morand, Jelena Trajkovic, Vincenzo Romei, Monika Harvey, Gregor Thut

**Affiliations:** ^1^Centre for Cognitive Neuroimaging, School of Psychology and Neuroscience, University of Glasgow, Glasgow, United Kingdom; ^2^School of Psychology, University of Nottingham, Nottingham, United Kingdom; ^3^Dipartimento di Psicologia, Centro Studi e Ricerche in Neuroscienze Cognitive, Alma Mater Studiorum – Università di Bologna, Bologna, Italy

**Keywords:** rhythmic TMS, visual perception, individual alpha frequency (IAF), alpha amplitude, subjective awareness

## Abstract

Alpha-band oscillatory activity over occipito-parietal areas is involved in shaping perceptual and cognitive processes, with a growing body of electroencephalographic (EEG) evidence indicating that pre-stimulus alpha-band amplitude relates to the subjective perceptual experience, but not to objective measures of visual task performance (discrimination accuracy). The primary aim of the present transcranial magnetic stimulation (TMS) study was to investigate whether causality can be established for this relationship, using rhythmic (alpha-band) TMS entrainment protocols. It was anticipated that pre-stimulus 10 Hz-TMS would induce changes in subjective awareness ratings but not accuracy, in the visual hemifield contralateral to TMS. To test this, we administered 10 Hz-TMS over the right intraparietal sulcus prior to visual stimulus presentation in 17 participants, while measuring their objective performance and subjective awareness in a visual discrimination task. Arrhythmic and 10 Hz sham-TMS served as control conditions (within-participant design). Resting EEG was used to record individual alpha frequency (IAF). A study conducted in parallel to ours with a similar design but reported after we completed data collection informed further, secondary analyses for a causal relationship between pre-stimulus alpha-frequency and discrimination accuracy. This was explored through a regression analysis between rhythmic-TMS alpha-pace relative to IAF and performance measures. Our results revealed that contrary to our primary expectation, pre-stimulus 10 Hz-TMS did not affect subjective measures of performance, nor accuracy, relative to control-TMS. This null result is in accord with a recent finding showing that for influencing subjective measures of performance, alpha-TMS needs to be applied post-stimulus. In addition, our secondary analysis showed that IAF was positively correlated with task accuracy across participants, and that 10 Hz-TMS effects on accuracy—but not awareness ratings—depended on IAF: The slower (or faster) the IAF, relative to the fixed 10 Hz TMS frequency, the stronger the TMS-induced performance improvement (or worsening), indicating that 10 Hz-TMS produced a gain (or a loss) in individual performance, directly depending on TMS-pace relative to IAF. In support of recent reports, this is evidence for alpha-frequency playing a causal role in perceptual sensitivity likely through regulating the speed of sensory sampling.

## Introduction

Perception of events in our surrounding environment does not only depend on stimulus features but also on endogenous factors such as those reflected in neuronal activity at baseline. In particular, EEG/MEG-research has linked pre-stimulus oscillatory activity in the alpha-band (8–14 Hz) to a dissociation between objective and subjective measures of visual perception. More specifically, it has been demonstrated that the amplitude of pre-stimulus alpha-oscillations is negatively correlated with decision confidence (Samaha et al., 2017) and perceptual awareness across different perceptuals tasks (Benwell et al., 2017, 2021), but that it is not related to task accuracy (Lange et al., 2013; Limbach and Corballis, 2016; Craddock et al., 2017; Iemi et al., 2017; Iemi and Busch, 2018; Kloosterman et al., 2019; see Samaha et al., 2020a for a review). Although most studies have demonstrated this relationship using low-level stimuli (e.g., Benwell et al., 2017; Samaha et al., 2017), Samaha et al. (2020b) have recently shown that the effect is robust also when higher-level visual areas are engaged, i.e., low pre-stimulus alpha-amplitude was associated with increased visibility ratings when participants were asked to discriminate between houses and faces. Experiments grounded in Signal Detection Theory (Green and Swets, 1966) have suggested that the relationship between spontaneous oscillations and subjective performance measures is due to a lowering of the pre-stimulus alpha activity being associated with a more liberal decision criterion, i.e., participants are more likely to report a stimulus as being present even when it is not, while their perceptual sensitivity to the target stimulus remains unchanged (Limbach and Corballis, 2016; Iemi et al., 2017). This can be explained by low pre-stimulus alpha-amplitude reflecting high excitability amplifying the representation of both signal and noise (Iemi et al., 2017; Zhou et al., 2021).

Although the correlative link between pre-stimulus alpha-amplitude and subjective visual awareness has been repeatedly demonstrated using EEG, whether pre-stimulus alpha-amplitude is causally modulating subjective confidence/awareness remains to be determined. The primary aim of our study was therefore to test for such a causal link. The relationship between a neural oscillator and behavior can be considered causal if (i) correlational EEG/MEG evidence links a frequency F(x) and a behavioral function X and (ii) non-invasive brain stimulation changes function X when applied at the frequency F(x), but not at a control frequency F(y) or an arrhythmic control condition (Thut et al., 2011). Here, we employed 10 Hz rhythmic-TMS over the right intraparietal sulcus (rIPS), while participants performed a luminance discrimination task, followed by single-trial ratings using the Perceptual Awareness Scale (PAS; Ramsøy and Overgaard, 2004). There is good evidence that TMS-induced and spontaneously generated modulations of alpha activity are comparable. Specifically, Thut et al. (2011), showed that TMS bursts tuned to the preferred frequency of the parietal cortex can promote alpha oscillations at the target site, which cycle at the natural frequency of the targeted generator. Furthermore, this TMS-induced narrow band alpha-amplitude increase varies as a function of the pre-TMS alpha-amplitude (shown for single-pulse TMS, Herring et al., 2015) as well as the pre-TMS alpha-phase (shown for TMS bursts, Thut et al., 2011). These two results indicate that TMS is interacting with and entraining a naturally occurring oscillation rather than imposing an artificial rhythm. Crucially, a complementary study by Romei et al. (2010) demonstrated that occipito-parietal TMS tuned to alpha frequency shapes perception in a way that is compatible with naturally occurring changes in alpha activity.

Based on the EEG/MEG literature reviewed above (e.g., Benwell et al., 2017; Samaha et al., 2017) and findings of rhythmic-TMS entrainment (see Romei et al., 2010; Thut et al., 2011), it was hypothesized that pre-stimulus 10 Hz-TMS over rIPS—relative to control TMS—should lead to a decrease in PAS ratings for stimuli in the contralateral left but not ipsilateral right hemifield, while accuracy on the task should remain unaffected. Yet, note that very recent work conducted in parallel to our study (Di Gregorio et al., 2022) could not find a causal relationship between pre-stimulus alpha-amplitude and awareness ratings using a pre-stimulus rhythmic-TMS entrainment protocol very similar to our approach.

In contrast to subjective visual awareness, Di Gregorio et al. (2022) found objective task accuracy to be predicted by pre-stimulus alpha-oscillations, namely their frequency, and established a causal relationship between pre-stimulus frequency and accuracy using rhythmic-TMS. Speeding up or slowing down the pre-stimulus alpha-oscillation via alpha-band TMS (at IAF + 1 Hz or IAF − 1 HZ, respectively) improved or worsened task accuracy, leaving subjective performance measures unaffected (Di Gregorio et al., 2022). This is in line with alpha-frequency playing a role in visual and/or attentional sampling (VanRullen and Koch, 2003; VanRullen, 2016; Kienitz et al., 2021). It shows that pre-stimulus alpha-pace may not only impact tasks relying on temporal resolution of visual perception such as temporal integration and segregation (Samaha and Postle, 2015; Wutz et al., 2018; Migliorati et al., 2020; for causal, mostly tACS-evidence see Cecere et al., 2015; Minami and Amano, 2017; Ronconi et al., 2018; Battaglini et al., 2020; Ghiani et al., 2021) but may also impact on perceptual accuracy more generally (Di Gregorio et al., 2022).

Therefore, following the report of pre-stimulus alpha-frequency accounting for accuracy (Di Gregorio et al., 2022), a secondary aim of our study (*post hoc* to its conception) was to examine whether our data would support such a relationship. To this end, we employed regression analysis. It was anticipated that resting IAF would be positively correlated with discrimination accuracy on the task, and that rhythmic-TMS alpha-pace (here 10 Hz) relative to IAF would affect accuracy but not subjective ratings, with IAF slower (or faster) than the fixed 10 Hz TMS frequency being associated with a TMS-induced improvement (or worsening) of task accuracy (as 10 Hz-TMS pace in these cases should speed up vs. slow down IAF).

## Materials and Methods

### Participants

A total of 21 participants were recruited for the study. All reported normal or corrected-to-normal vision, no history of neurological or psychiatric disorders, and no contraindication to brain stimulation, according to the TMS screening questionnaire (Rossi et al., 2009). They all gave written informed consent before the start of the experiment. The study was approved by the Ethics Committee of the College of Science and Engineering at the University of Glasgow and was conducted in accordance with the latest revision of the Declaration of Helsinki. Data from 4 participants were excluded from the analysis because their individual alpha peak frequency could not be identified (see below: EEG recording and alpha peak frequency identification). Thus, 17 participants were included in the final sample (12 females, mean age = 23 years, age range: 19–36 years, right-handedness: 16/17).

Sample size was determined based on previous literature. Specifically, previous TMS studies on oscillatory entrainment had considered a sample size between 7 and 17 participants (Sauseng et al., 2009; Romei et al., 2010, 2011, 2012, 2016; Thut et al., 2011; Chanes et al., 2013; Albouy et al., 2017; Vernet et al., 2019). In addition, *post hoc* power analysis (G-power 3.1) revealed that, for all rmANOVA and regression analyses used in our study, values of Power (1-b err prob) are > 0.95, for the previously obtained effect size (Di Gregorio et al., 2022, η*_*p*_*^2^ = 0.302).

### Experimental Procedure

The experiment consisted of two sessions of maximally 2 h each, 48 h apart at least. Participants sat in a dimly lit testing room in front of an LCD monitor (resolution 2,560 × 1,440, refresh rate 100 Hz, viewing distance 70 cm), with their heads stabilized in a chin rest. In the first session, participants underwent a threshold titration procedure and completed the first experimental block. The second session consisted of a threshold re-assessment and performing the remaining blocks of the experimental task.

### Experimental Task and Visual Stimuli

The current work was designed as a follow-up to Benwell et al. (2017), who demonstrated a correlative relationship between pre-stimulus alpha amplitude and PAS ratings but not accuracy. Therefore, the experimental task design remained the same, with the addition of the TMS entrainment protocol. The task used here (and in Benwell et al., 2017) can be defined as a 2-AFC discrimination task, as it involves the luminance discrimination of circular patches (brighter vs. darker) from the background. The patches were created with a Gaussian envelope (size = 1.3°) and were presented on a gray background (RGB: 127, 127, 127) in the lower left or right visual field (VF = 3.7° vertical and ± 4.1° horizontal eccentricity). Before the experimental task, the luminance of the Gaussian patches was individually adjusted to obtain four contrast levels (two lighter and two darker than the gray background, one for each side of the VF) by using a threshold assessment procedure. The contrast of the stimuli presented varied from 0.011 to 0.05% of the maximal contrast of the gray patches.

### Threshold Titration

The aim of the titration session was to identify four contrast values (resulting in four luminance levels: lighter-than-background patch—left VF, lighter-than-background patch—right VF, darker-than-background patch—left VF, darker-than-background patch—right VF) corresponding to a detection rate of 80%. The thresholds were identified using the method of constant stimuli (Urban, 1910). On the first day of testing, at the beginning of the session, 10 evenly spaced contrast values ranging from 0.011 to 0.05% of the maximal contrast of the light/dark patches were presented in a randomized order, in the lower left or right visual field (see Stimuli for details). The first stage included a total of 120 trials per participant, with all contrast values being tested 12 times (3 trials per stimulus condition). On each trial, there was a brief (150 ms, 1,000 Hz) warning tone followed by a 1,000 ms interval, after which the stimulus appeared on the screen for 30 ms. Participants were asked to keep their eyes on a central fixation cross (size = 0.5°) and to judge the brightness of the stimulus relative to the gray background, by pressing two buttons on the numeric keyboard (“1” for darker, “2” for lighter stimuli), using their right index and middle fingers. They were required to make a guess on the trials in which they did not see the stimulus. At the end of this block, a sigmoid function was fitted to the data to identify the first contrast value at which participants’ performance was at ceiling (i.e., 100% accuracy). Participants were then tested again in four blocks but with stimulus contrasts now ranging from the lowest contrast value (i.e., 0.011%) to the newly identified contrast value of maximal performance below ceiling. These blocks included 10 trials for each contrast and stimulus type, resulting in a total number of 400 trials per participant. At the end of this block, sigmoid functions were again fitted to the data for both light and dark stimuli for each visual field (i.e., left and right). While in Benwell et al. (2017), six contrast values were used (yielding six luminance levels: three light and three dark patches) corresponding to 25, 50, and 75% of correct detection performance, here contrast values yielding detection thresholds of 80% were extracted for each participant for each of the four stimulus conditions: lighter-than-background patch left VF, lighter-than-background patch right VF, darker-than-background patch left VF, darker-than-background patch right VF. *T*-tests on the contrast values for lighter/darker stimuli between the left and right visual hemifield showed no differences between the hemifields [*t*(16) = 1.26, *p* = 0.22].

On the second day of testing, a shorter threshold re-assessment was performed to verify whether participants’ performance was similar to that of the previous session. Ten evenly spaced contrasts ranging from previously identified contrast levels corresponding to 50 and 100% detection accuracy were tested in a total of 240 trials (10 contrasts × 4 stimulus conditions × 6 trials). Sigmoid functions were once again fit to the data for each of the stimulus conditions to ensure that the 80% detection threshold was confirmed and was consistent with that from the first session.

### Discrimination Task

**FIGURE 1:**
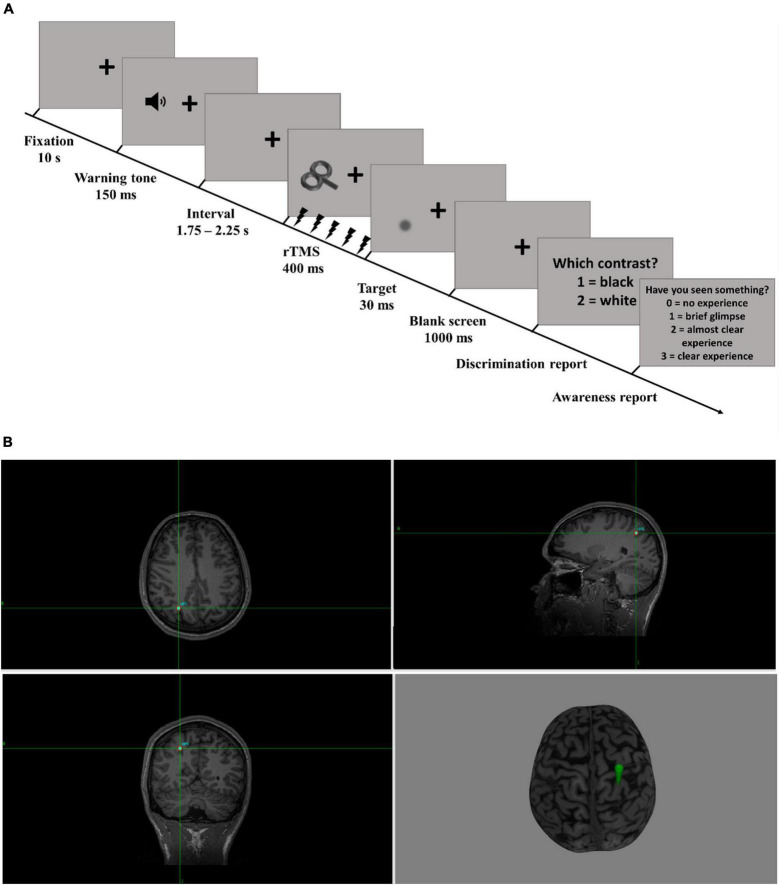
Task design. **(A)** Trial time course. The trials started with the presentation of a fixation cross for 10 s. A tone warned participants that the target stimulus would be presented shortly. After an interval ranging from 1.75 to 2.25 s, rhythmic-TMS was applied in one of the three experimental conditions (rhythmic, arrhythmic, or sham). The target stimulus was presented at the end of the last TMS pulse for 30 ms. Participants were then prompted to indicate the stimulus luminance of the target relative to the background and rate their subjective perceptual experience on the PAS. **(B)** TMS target. This MR scan from a representative participant illustrates the target that was used for the neuro-navigation (right intraparietal cortex, rIPS). The TMS coil was oriented such that its handle was pointing upwards (R, Right; L, Left; A, Anterior; P, Posterior).

The experimental task was a luminance discrimination task. Each trial (see Figure 1A) started with a black fixation cross for 10 s, followed by a warning tone (150 ms, 1,000 Hz). After a randomized interval that could range from 1.75 to 2.25 s, rhythmic-TMS was applied for 400 ms (5 pulses). Stimulus onset was synchronized with the last TMS pulse. The stimulus, consisting of a light/dark gray Gaussian patch, appeared on the screen for 30 ms (3 frames) in the lower visual field, either to the left or to the right. A blank, fixation cross screen then followed for 1,000 ms, after which participants were prompted to judge the brightness of the stimulus relative to the gray background, pressing with their right hand “1” on the numeric keyboard for stimuli darker than the background, and “2” for lighter stimuli. They were asked to make a guess on trials in which they did not perceive any stimulus. After the button press, another question appeared on the screen, prompting participants to rate the clarity of their perception on the four-point Perceptual Awareness Scale (PAS; Ramsøy and Overgaard, 2004). The four PAS categories were: (0) “no experience,” (1) “brief glimpse,” (2) “almost clear experience,” and (3) “clear experience” of the stimulus. Responses were given by pressing four different buttons on the numeric pad of the keyboard (“0,” “1,” “2,” “3”). The experimental task was divided into 5 blocks. Each block was composed of 60 trials: 5 trials at the individually adjusted stimulus contrast for each of the four stimulus conditions per each of the three TMS conditions (i.e., rhythmic TMS, arrhythmic TMS, sham TMS). This yielded a total of 300 trials per participant, with the order of the trials being randomized across each block. Participants had a self-paced break after 30 trials, and at the end of each block. The threshold assessment and the behavioral task were programmed and run in MATLAB (MathWorks Inc.), using the Psychophysics Toolbox Version 3 functions (Kleiner et al., 2007).

### Transcranial Magnetic Stimulation Site and Neuro-Navigation

The TMS target site over the right intraparietal sulcus (rIPS; Talairach coordinates: 28, −51, 50) was adopted from a previous study reporting effects of pre-stimulus 10 Hz-TMS on visual detection performance with both parietal and occipital TMS (Romei et al., 2010). We decided on stimulating the parietal rather than occipital cortex because occipital TMS is more likely to induce phosphenes and therefore to interfere with visual processing. Furthermore, Romei et al. (2010) reported statistically indistinguishable effects on perception across IPS and occipital 10 Hz-TMS, speaking in favor of a common parieto-occipital alpha mechanism. As anticipated, no participant reported having perceived phosphenes during the execution of the task.

To be eligible for the study, all participants had to have an individual high-resolution T1-weighted anatomical MRI scan acquired at the Centre for Cognitive Neuroimaging (CCNi) at the University of Glasgow. The MRI scans were recorded in a 3T MR scanner (Magnetom Trio Siemens, Erlangen, Germany) using a 3D magnetization prepared rapid acquisition gradient echo sequence (ADNI- MPRAGE) (192 axial slices; voxel size = 1 × 1 × 1 mm; TR = 1,900 ms; TE = 2.52 ms; inversion time = 900 ms; slice thickness = 1 mm; FoV = 256 mm; image resolution = 256 × 256; excitation angle = 9°). rIPS coordinates were first projected on each individual reconstructed 3D anatomical MRI scan using Brainsight (Rogue Research) (see Figure 1B for an example). The MRI scans were normalized into standard Talairach space to identify the rIPS coordinates and then projected into native space. The anatomical MRI scans were co-registered with the participant’s head, and the TMS coil was then neuro-navigated to the target site.

### Transcranial Magnetic Stimulation Intensity and Conditions

TMS was applied at a fixed intensity of 65% of the maximum stimulator output (MSO) using a Magstim Rapid2 Transcranial Magnetic Stimulator via a 70 mm figure-of-eight coil (Magstim Company). Three TMS conditions were run per participant. In all conditions, the rIPS was stimulated with short TMS bursts (five pulses). For the active (rhythmic) TMS, the stimulation was set at an alpha frequency of 10 Hz (10 Hz-TMS). The coil was oriented with the handle pointing upwards (along the sagittal plane) so that the center of the coil was overlaying the rIPS in each individual anatomical MRI scan, with the TMS coil inducing currents perpendicular to the target gyrus in most participants [maximizing TMS efficacy (Thielscher et al., 2011; Thut et al., 2011)]. Additionally, two control conditions were run. In one control—arrhythmic TMS (ar-TMS)—the same number of TMS pulses was applied as for 10 Hz-TMS (also within the same time window), but with randomized inter-pulse intervals of 70, 80, 120, and 130 ms, respectively. This control was intended to determine if a behavioral effect was due to alpha entrainment or a basic response to rapid-rate TMS bursts. Lastly, for the sham condition (10 Hz-TMS sham), a second TMS coil (also a 70 mm figure-of-eight coil, 65% intensity of MSO) was positioned over the main coil/target area but perpendicular to the surface of the participant’s head. This emulated the sound clicks associated with the 10 Hz-TMS, while the current was discharged away from the cortex, thus helping to account for non-specific effects of TMS. The experimental trials were randomized, with participants receiving all three TMS conditions in every block.

### Electroencephalographic Recording and Alpha Peak Frequency Identification

In the first session only, resting EEG was recorded for 2 min with eyes open and 2 min with eyes closed employing a BrainAmp system (Brain Products, GmbH, Munich, Germany—BrainVision Recorder) using a cap with 3 Ag/AgCl pellet pin electrodes (EasyCap GmbH, Herrsching, Germany). Electrodes were placed according to the 10-10 International System at locations O1, Oz, and O2. Two extra electrodes served as ground (TP9) and online references (Cz). Electrode impedances were kept below 10 kΩ.

Individual alpha peak frequency was estimated per participant from the occipital electrodes (O1, Oz, and O2) using the eyes-closed data. To determine the individual alpha peak frequency, an automated estimation process was adopted (Corcoran et al., 2018). Pre-processing steps were performed using Brain Vision Analyzer 2.0 (Brain Products). The automated method of Corcoran et al. (2018) first extracts the power spectral density (PSD) of the pre-processed data and applies the Savitzky-Golay filter (SGF; Savitzky and Golay, 1964) to smooth the PSD function. Then, the first and second-order derivatives are calculated and analyzed for a distinctive spectral peak in the alpha-band (8–14 Hz). To qualify as a valid peak, the largest peak detected has to be at least 20% higher than any other peak within the alpha band, and to have the highest power value at least 1 standard deviation from the PSD mean. Overall, 17 out of 21 participants met these conditions and were included in the final analyses.

### Statistical Analysis

Trials with extreme reaction times were removed based on the median ± 1.5 * interquartile range (IQR) criterion (Tukey, 1977). Participants had to have a minimum of 75 trials per TMS condition to be included in the final sample, which was met by all final 17 participants.

Primary hypothesis/analyses: It was expected that a behavioral effect would be present during 10 Hz-TMS as compared to both control conditions (ar-TMS, 10 Hz-TMS_sham_) for PAS ratings but not for accuracy, in the visual field contralateral to the stimulated hemisphere. To test this, a within-subjects, repeated-measures analysis of variance (rmANOVA) was conducted on the two behavioral measures (accuracy and PAS) with the factors TMS condition (10 Hz-TMS vs. ar-TMS vs. 10 Hz-TMS_sham_) × Target Location (left vs. right visual field). Discrimination accuracy and awareness ratings were analyzed separately.

Secondary hypotheses/analyses: It was expected that IAF would positively correlate with task accuracy and that rhythmic TMS (as opposed to arrhythmic TMS) would influence this relationship depending on IAF and hemifield. In contrast, no relationship of IAF with PAS was anticipated. To test this, multiple regression models were run, with the factors TMS condition, hemifield and IAF used as predictors of behavioral outcome (i.e., accuracy and PAS ratings). For further exploration, Pearson’s correlations were used to test the specificity of 10 Hz-TMS effects relative to ar-TMS in the hemifield contralateral to the stimulation site (in relation to the individual factor IAF). Statistical analyses were performed using RStudio 3.4.1 (R Core Team, 2020).

## Results

Participants were presented with visual stimuli at threshold levels. After stimulus presentation, they had to first discriminate the stimulus from the background yielding a measure of performance accuracy. Then, participants were asked to rate their subjective awareness of the stimulus on the PAS scale (Figure 1A).

### Overall Task Performance

**FIGURE 2:**
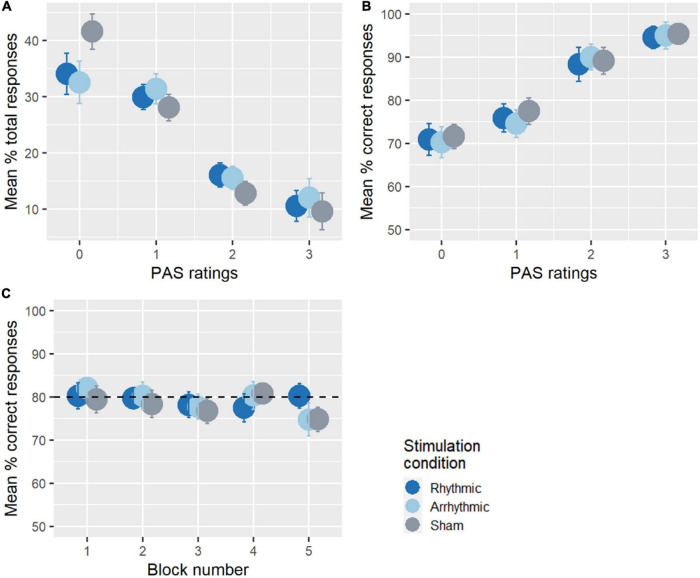
Overall task performance. **(A)** The use of the PAS ratings across the experiment. On average, participants reported having more “no experience” (PAS = 0) or “brief glimpse” (PAS = 1) than “almost clear experience” (PAS = 2) or “clear experience” (PAS = 3) of the stimulus. **(B)** Average percentage of correct responses as a function of the PAS ratings. The accuracy of the participants increased with awareness. **(C)** Accuracy across experimental blocks. There was no significant difference between accuracy across the blocks during the 10 Hz-TMS sham condition. The performance of the participants was around 80% of their detection threshold throughout the entire experimental session, indicating that stimulus-titration was successful.

Figure 2A illustrates how participants used the PAS scale. On average, participants reported having “no experience” of the stimulus in 36.1% of all trials, “brief glimpse” in 29.8%, “almost clear experience” in 14.8%, and “clear experience” in 10.7% of trials. PAS scores for each target location are reported in Supplementary Figure 1. Accuracy was analyzed as a function of the clarity of the subjective experience (Figure 2B). This indicated that as the clarity of the target stimulus increased, so did accuracy, ranging from 70.9% when participants reported having “no experience” of the target stimulus, 76% when rating “brief glimpse,” 89.2% when rating “almost clear experience,” to 95% when the perceptual experience was “clear.” Next, we checked whether the detection threshold manipulation had been successful and whether the performance across blocks remained at the target levels (80% of the detection threshold of each participant, sham data considered, see Figure 2C). To this end, we ran a repeated-measures ANOVA on discrimination accuracy in the 10 Hz-TMS sham condition across each experimental block. There was no significant difference between accuracy levels in the five experimental blocks [*F*_(4, 64)_ = 0.63, *p* = 0.63, $\eta_{G}^{2}$ = 0.03], with the average detection threshold ranging from 74 to 80% across the experiment.

### No Specific Effects of Pre-stimulus 10 Hz-Transcranial Magnetic Stimulation, Relative to Control Transcranial Magnetic Stimulation (Arrhythmic TMS, 10 Hz-TMS_sham_), on Accuracy vs. Perceptual Awareness Scale Ratings

To probe for potential, differential effects of pre-stimulus 10 Hz-TMS on objective measures of visual task performance vs. subjective measures of perceptual experience, we ran a two-way repeated-measures ANOVAs with the factors TMS condition and target location for each measure (accuracy and PAS ratings).

#### Accuracy

**FIGURE 3:**
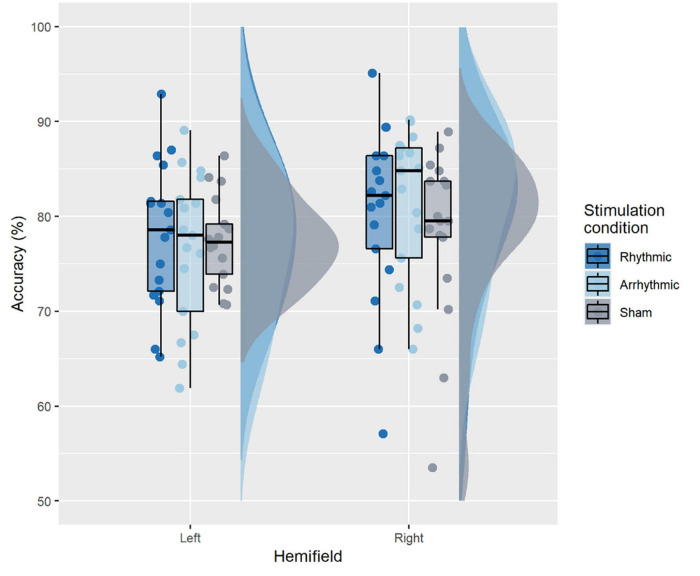
Accuracy across conditions. The boxplots show a representation of the median and the first and third quartiles of the average accuracy per TMS condition and target location. The whiskers of the boxplot can take a maximal value up to 1.5*interquartile range. The boxplots are superimposed with individual data points, while the clouds represent the probability distribution of the sample.

Figure 3 illustrates accuracy across conditions. The 2-way rmANOVA revealed no significant main effects of TMS condition [*F*_(2, 32)_ = 0.65, *p* = 0.53, $\eta_{G}^{2}$ = 0.006], nor target location [*F*_(1, 16)_ = 2, *p* = 0.17, $\eta_{G}^{2}$ = 0.028] and there was no significant interaction between TMS condition and target location [*F*_(2, 32)_ = 1.23, *p* = 0.305, $\eta_{G}^{2}$ = 0.010].

#### Perceptual Awareness Scale Ratings

**FIGURE 4:**
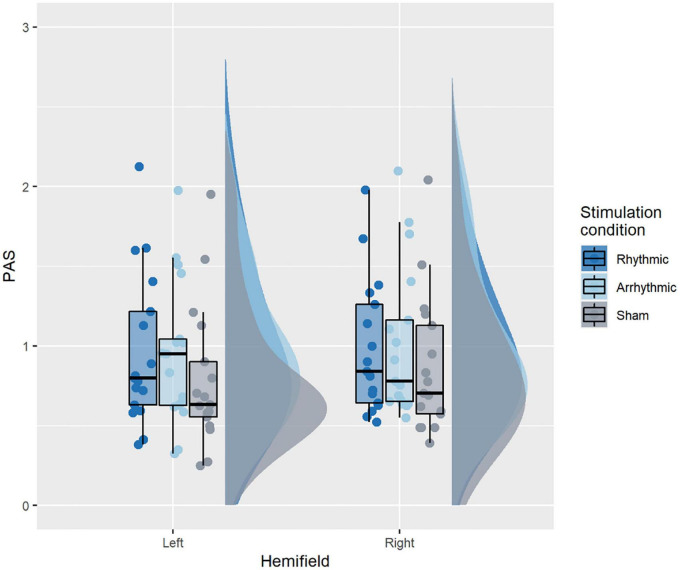
PAS ratings across conditions. A significant main effect of TMS condition was found, explained by active TMS (10 Hz-TMS, ar-TMS) improving PAS ratings relative to 10 Hz-TMSsham. The boxplots show a representation of the median and the first and third quartiles of the average PAS ratings per TMS condition and hemifield. The whiskers of the boxplot can take a maximal value up to 1.5*interquartile range. The boxplots are superimposed with individual data points, while the clouds represent the probability distribution of the sample.

Figure 4 illustrates PAS ratings across conditions. The corresponding rmANOVA revealed a significant main effect of TMS condition [*F*_(2, 32)_ = 16.4, *p* < 0.001, $\eta_{G}^{2}$ = 0.02]. PAS ratings were significantly higher during 10 Hz-TMS trials (*M* = 0.979) than during 10 Hz-TMS_sham_ trials (*M* = 0.828) [*t*(16) = 4.27, *p* = 0.001, *r*^2^ = 0.46, Bonferroni corrected], and during ar-TMS trials (*M* = 0.983) as compared to 10 Hz-TMS_sham_ trials [*t*(16) = 4.53, *p* = 0.001, *r*^2^ = 0.48, Bonferroni corrected]. No significant difference was observed for PAS ratings between 10 Hz-TMS and ar-TMS [*t*(16) = −0.37, *p* = 1, *r*^2^ = 0.04, Bonferroni corrected]. There was no significant main effect of hemifield [*F*_(1, 16)_ = 0.64, *p* = 0.43, $\eta_{G}^{2}$ = 0.003] and no significant interaction between TMS condition and hemifield [*F*_(2, 32)_ = 0.96, *p* = 0.4, $\eta_{G}^{2}$ = 0.0008].

The finding that all effects of TMS condition were driven by active TMS bursts (10 Hz-TMS and/or ar-TMS) relative to sham, but without any significant difference between 10 Hz-TMS and ar-TMS, indicates that TMS effects on PAS ratings were not due to 10 Hz-entrainment but reflect an unspecific, likely alerting response to the stronger peripheral stimulation of the active TMS bursts relative to sham.

### Pre-stimulus 10 Hz Transcranial Magnetic Stimulation Pace Relative to Individual Alpha Peak Frequency Modulates Accuracy but Not Perceptual Awareness Scale Ratings

To test whether pre-stimulus alpha-frequency is related to visual accuracy and/or subjective awareness, we ran multiple regression analyses to examine whether accuracy and/or PAS ratings, respectively, could be predicted by IAF, rhythmic-TMS alpha pace relative to IAF, TMS condition and hemifield.

#### Accuracy

**FIGURE 5:**
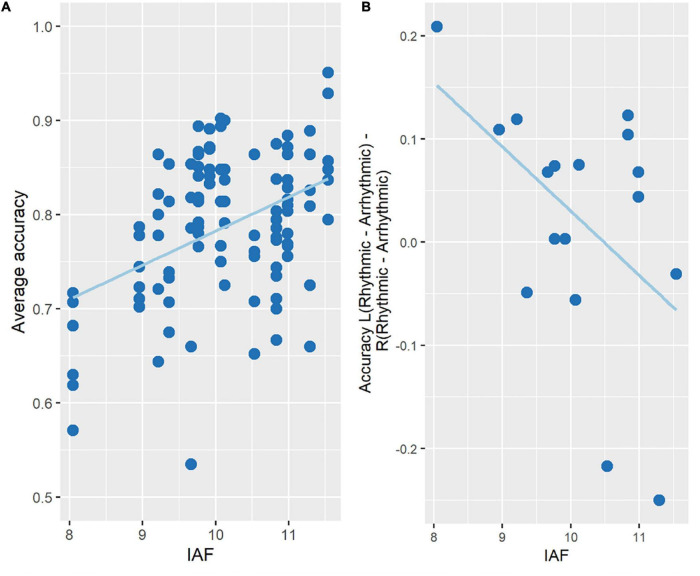
Change in accuracy as a function of IAF. **(A)** A multiple regression analysis showed that accuracy increased with increasing IAF measured at rest across participants. The figure plots the experimental conditions (3 TMS conditions **×** 2 target locations) for each participant. **(B)** Spatially specific effects (LVF vs. RVF) of entrainment (10 Hz-TMS minus ar-TMS) as a function of IAF. Participants with lower IAF had accuracy increased by 10 Hz-TMS in the hemifield contralateral to the TMS site, hence *benefitting* from the 10 Hz-TMS stimulation, while the opposite effect can be seen in participants with high IAF.

For accuracy, the regression model was statistically significant [*F*_(4_, _97)_ = 6.203, *p* < 0.001, *R*^2^ = 0.17], with the variable IAF significantly adding to the prediction (*p* < 0.01). More specifically, accuracy increased with increasing IAF across participants (Figure 5A).

To further explore whether rhythmic TMS (as opposed to arrhythmic TMS) may have influenced this relationship, we examined whether the effect of 10 Hz-TMS on accuracy (relative to ar-TMS) depended on the offset of 10 Hz from IAF. With entrainment, one would expect 10 Hz-TMS to speed up the IAF and hence increase accuracy the more the participant’s IAF is in the lower alpha range (< < 10 Hz). Conversely, 10 Hz-TMS should slow down the IAF and hence decrease accuracy the more the participant’s IAF is in the higher alpha range (> > 10 Hz). This effect should be found for stimuli contra- but not ipsilateral to the stimulated hemisphere. To explore this, spatially specific entrainment effects of 10 Hz-TMS on accuracy were estimated by the following subtraction term:

Accuracy _Left Hemifield_ (10 Hz-TMS—ar-TMS)—Accuracy _Right Hemifield_ (10 Hz-TMS—ar-TMS), where left/right hemifield is contra-/ipsilateral to the TMS site, and entrainment effects are inferred by subtracting ar-TMS from 10 Hz-TMS. The relationship between this behavioral measure and the IAF was then examined using the Bootstrapped Pearson’s correlation from the Robust Correlation Toolbox in Matlab (Pernet et al., 2013). This revealed a significant negative correlation (Pearson’s *r* = −0.485, *p* < 0.05, CI = [−0.75, −0.0003]), showing that as IAF decreased across participants, right-hemispheric 10 Hz-TMS improved left hemifield accuracy (as compared to the control TMS conditions) (see Figure 5B and Supplementary Figure 2). We regard this Bootstrapped Pearson correlation as meaningful given prior results from a recent alpha-TMS entrainment study (see Di Gregorio et al., 2022): namely of an equivalent linear relationship, whereby effects on pre-stimulus alpha-frequency and objective visual task performance co-varied after entrainment with pre-stimulus 10 ± 1 Hz-TMS as compared to sham 10 ± 1 Hz-TMS (Figure 4B in Di Gregorio et al., 2022).

#### Perceptual Awareness Scale Ratings

For the PAS ratings, the multiple regression model including the variable IAF was not significant, and hence the model was not further investigated.

## Discussion

In the present study, we aimed to test for a causal relationship between alpha-oscillations and subjective measures of performance, by using rhythmic TMS in the alpha-band to modulate perceptual awareness. Based on recent findings in the literature and prior rhythmic-TMS entrainment evidence, we expected pre-stimulus 10 Hz-TMS over the rIPS to cause PAS ratings to decrease in the contralateral left, but not ipsilateral right hemifield. This hypothesis was not confirmed, as there was no significant interaction between TMS condition (10 Hz-TMS vs. controls) and hemifield (left vs. right). Although we observed significant effects of TMS (for PAS ratings), these were all unspecific as they were present in both active TMS conditions (10 Hz-TMS and arrhythmic TMS) relative to sham 10 Hz-TMS. Hence, these effects of TMS are best explained by alerting responses to the more salient active TMS bursts (rhythmic and arrhythmic). The secondary hypothesis of the present study was for resting individual alpha peak frequency (IAF) to be positively correlated with discrimination accuracy, and for this relationship to be influenced depending on rhythmic-TMS alpha-pace offset from IAF. Indeed, we found that faster IAF was associated with higher discrimination accuracy. Furthermore, TMS-probing of this relationship confirmed causality. Although the evidence was moderate to weak (*r* = 0.48), we consider this meaningful, given its accordance with prior evidence by Di Gregorio et al. (2022), hence suggesting that the speed of pre-stimulus alpha-oscillations causally modulates the participants’ visual sensitivity to a target stimulus.

Our null result as to a causal link between pre-stimulus alpha-activity and subjective awareness ratings further replicates findings by Di Gregorio et al. (2022) employing a pre-stimulus alpha-TMS design equivalent to ours (see their experiment 2). Similar to our study, their design consisted of 5-pulse TMS bursts applied immediately prior to target onset over the right occipital cortex at individual alpha-band frequency (IAF), while participants performed a visual detection task and rated their perceptual confidence upon a delayed confidence prompt. Similar to the present findings, it was reported that rhythmic alpha-band TMS, relative to sham TMS bursts, did not affect perceptual confidence levels (nor accuracy), even though evidence of entrainment by rhythmic-TMS was found in the EEG activity of participants (Di Gregorio et al., 2022). Di Gregorio et al. (2022) argued that rather than speaking against a causal relationship between alpha-amplitude and confidence ratings, this negative finding may have been explained by the short-lived nature of the pre-stimulus rhythmic-TMS entrainment effects, wearing off long before the delayed confidence judgment had to be given (several seconds after TMS offset/stimulus presentation). This may also apply to our study, as in analogy to Di Gregorio et al. (2022), we presented the awareness prompt > 1 s after rhythmic-TMS offset/stimulus onset (see Figure 1A). Di Gregorio et al. (2022) then tested, in a follow-up experiment, whether the timing of the TMS burst delivery—relative to the confidence rating—is key, by delivering the TMS bursts before the confidence prompt instead of pre-stimulus. This indeed affected the subjective evaluation of performance (but not accuracy) in the expected direction, such that higher TMS-induced alpha-amplitudes prior to the prompt were associated with lower confidence. This appears to be in keeping with recent correlational evidence from the literature, across different tasks (Benwell et al., 2017, 2021; Samaha et al., 2017; Wöstmann et al., 2019). However, Di Gregorio et al. (2022) found the causal relationship between alpha-amplitude and subjective evaluation of performance for meta-d’ (Maniscalco and Lau, 2012), not raw confidence scores, measuring how well subjective confidence judgments can distinguish between correct and incorrect decisions, and hence indicating that alpha-amplitude plays a role in post-perceptual metacognitive decision making (Murphy et al., 2015; Fleming and Daw, 2017; Pereira et al., 2020). In a similar vein, but using transcranial alternating current stimulation (tACS) to affect somatosensation, Craddock et al. (2019) found 10 Hz-tACS to increase the reports of a stimulus being present even when it was not there, consistent with the hypothesis that alpha-oscillations lead to a more liberal decision criterion (Iemi et al., 2017). In brief, since in the present study the participants’ confidence responses were required more than 1 s after TMS offset and given the short-lived nature of rhythmic-TMS entrainment, our null results might be explained by a suboptimal timing of the PAS rating prompt, relative to TMS. Future research could study this by asking participants to give the PAS ratings immediately after stimulus presentation, and examining how this is impacted by pre-stimulus rhythmic-TMS in the alpha-band. See also Hobot et al. (2020) who demonstrated that perceptual awareness, but not accuracy, was successfully modulated via a single TMS pulse delivered to the primary motor cortex under this condition. Alternatively, our null results as to the 10 Hz-TMS effects on PAS scores may be explained by low overall PAS scores, due to a more conservative bias being induced because of the twofold nature of our task (discrimination and subsequent PAS rating), rather than asking for PAS ratings only. This could have led to a floor effect, preventing us from capturing TMS-effects. Although we cannot completely discard this possibility, the same task when used before revealed a correlative relationship between pre-stimulus alpha amplitude and PAS ratings (Benwell et al., 2017), making this alternative less likely.

Our findings that rhythmic-TMS alpha-pace relative to IAF is impacting accuracy but not confidence ratings are in agreement with the work of Di Gregorio et al. (2022), who reported that the trial-by-trial variability in pre-stimulus EEG alpha-frequency predicted task accuracy, and that this relationship was causally modulated using rhythmic-TMS at IAF ± 1 Hz, improving/impairing accuracy, respectively. It is worth mentioning that the task choice was different across our study and Di Gregorio et al. (2022), where peri-threshold stimulus detection was used. However, the finding of alpha-frequency in shaping perceptual sensitivity is converging, speaking in favor of its role in shaping perceptual accuracy across different perceptual tasks. Similarly, here we stimulate IPS as opposed to occipital areas having been stimulated in Di Gregorio et al. (2022), thus it would seem that the role of alpha-frequency is not only generalized across different task, but also that it’s not strictly limited to the occipital brain regions. Although our choice of stimulating IPS was mostly based on avoiding phosphenes, a previous study has shown that effects of 10 Hz-TMS over IPS and occipital cortex on visual tasks are statistically indistinguishable (Romei et al., 2010). In the present study, rhythmic IPS-TMS at alpha frequency may have influenced luminance discrimination by affecting attentional sampling (e.g., Kienitz et al., 2021), or by exerting its effects indirectly on lower level visual areas through downstream connections.

Until very recently, the focus in the literature as to the perceptual role of alpha-pace was on its perceptual framing potential, where faster IAF translates into better temporal sensitivity/resolution in the visual domain (Samaha and Postle, 2015; Minami and Amano, 2017; Ronconi et al., 2018; Wutz et al., 2018; Battaglini et al., 2020), or into a narrower temporal window of perception with multisensory processing (Cecere et al., 2015; Keil and Senkowski, 2017; Cooke et al., 2019; Bastiaansen et al., 2020; Migliorati et al., 2020; Venskus and Hughes, 2021; Venskus et al., 2021; Noguchi, 2022). However, as recently demonstrated (Di Gregorio et al., 2022) and confirmed here, IAF modulates visual sensitivity even in non-temporal tasks. Therefore, it seems that the length of one alpha cycle not only influences the perceptual segregation between distinct cycles, but also the amount of processing abilities within a cycle, with a more effective sampling per cycle for higher than lower alpha frequencies. Or in other words, while higher frequency is expected to aid temporal resolution by creating more sampling frames per second, recent and our data suggest that at the same time, higher frequency also means that less time is employed to create a single sampling frame, leading to higher processing capacities. This accords with EEG/MEG results indicating that faster IAF helps individuals to adjust to increasing task demands in cognitive (Haegens et al., 2014; Maurer et al., 2015) and physical tasks (Gutmann et al., 2015; Hülsdünker et al., 2016). In addition, Jann et al. (2010) demonstrated that participants with higher IAF had an increased regional cerebral blood flow in areas associated with attention modulation and preparedness for external input. Mechanistically, based on computational models in real and artificial neural networks, Cohen (2014) infers that changes in IAF encode information about input intensity and play a role in spike timing variability, such that higher IAF will cause neurons to fire at higher input levels, thus enabling more accurate responses. Mierau et al. (2017) hypothesize that IAF fluctuations form an adaptive mechanism that mirrors the activation level of underlying neuronal populations, with fast frequencies facilitating sensory sampling, neuronal computation, and communication between brain regions. By extension, faster alpha-paces are expected to be associated with heightened processing resources and therefore more accurate task performance, as demonstrated here and in prior research.

## Conclusion

While failing to demonstrate an effect of pre-stimulus 10 Hz-TMS on perceptual awareness rating (compared to arrhythmic and sham TMS), we found individual alpha peak frequency (IAF) to be positively correlated with task accuracy and this relationship to be modulated by pre-stimulus rhythmic TMS in the alpha-band. Together with prior, equivalent evidence from Di Gregorio et al. (2022), our results support a functional, causal role of alpha-frequency in perceptual sensitivity.

## Data Availability Statement

The raw data supporting the conclusions of this article will be made available by the authors, without undue reservation.

## Ethics Statement

The studies involving human participants were reviewed and approved by the Ethics Committee of the College of Science and Engineering at the University of Glasgow. The patients/participants provided their written informed consent to participate in this study.

## Author Contributions

AC, DV, SM, MH, and GT conceived the project and designed the experiment. AC, DV, and SM implemented the experiment. AC conducted the experiment. AC, JT, and VR analyzed data. AC, MH, and GT wrote the first draft of the manuscript. AC, DV, SM, JT, VR, MH, and GT contributed to the final draft of the manuscript. All authors contributed to the article and approved the submitted version.

## Conflict of Interest

The authors declare that the research was conducted in the absence of any commercial or financial relationships that could be construed as a potential conflict of interest. The reviewer CT declared a past collaboration with one of the authors, GT to the handling editor.

## Publisher’s Note

All claims expressed in this article are solely those of the authors and do not necessarily represent those of their affiliated organizations, or those of the publisher, the editors and the reviewers. Any product that may be evaluated in this article, or claim that may be made by its manufacturer, is not guaranteed or endorsed by the publisher.
