# Supplementary material for: Effects of Rhythmic Transcranial Magnetic Stimulation in the Alpha-Band on Visual Perception Depend on Deviation From Alpha-Peak Frequency: Faster Relative Transcranial Magnetic Stimulation Alpha-Pace Improves Performance
Source: Front Neurosci. 2022 Jun 17;16:886342. doi: 10.3389/fnins.2022.886342 (PMC9247279; doi:10.3389/fnins.2022.886342)
Supplement: Supplementary file 1 [file Image_1.PDF]

Supplemental material

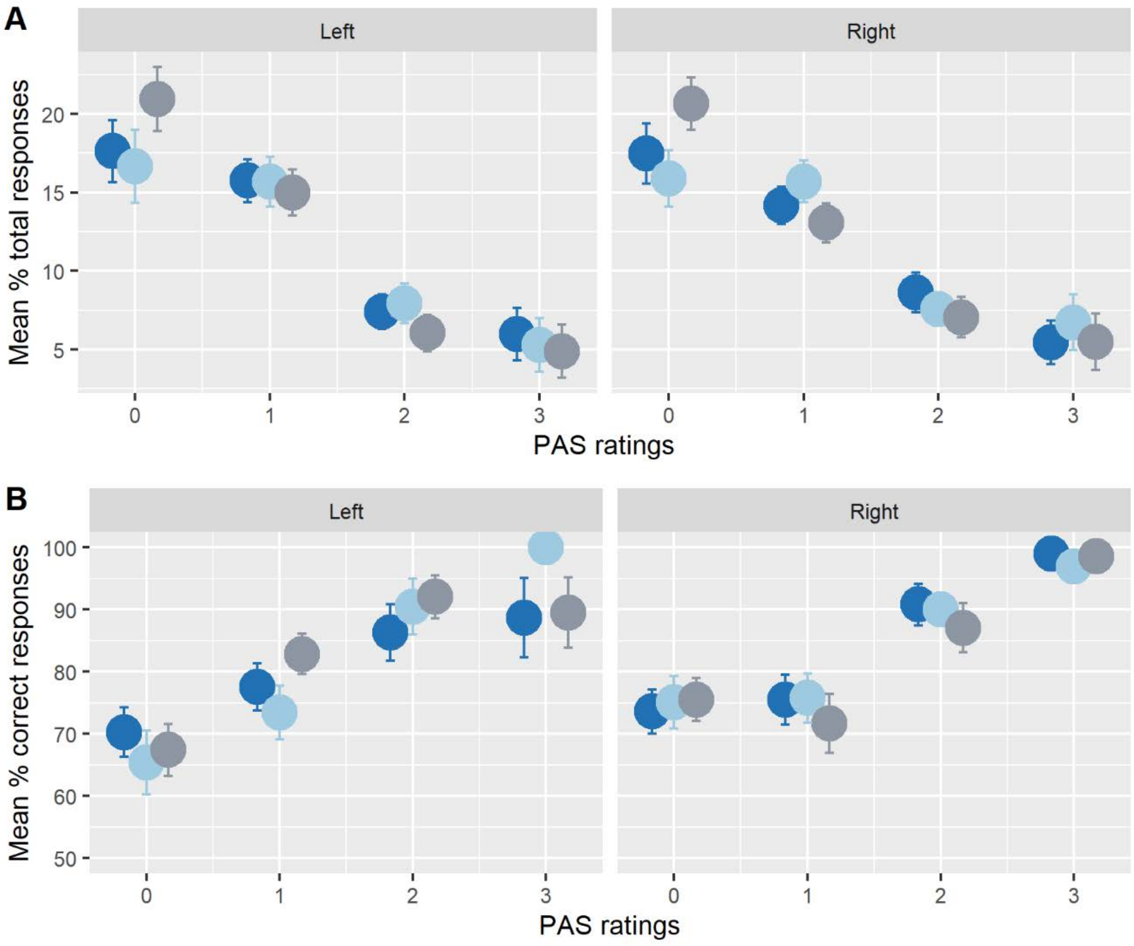

Supplementary Figure 1: Overall task performance. (A) The use of the PAS ratings across the experiment, separated by target location. On average, participants reported having more often "no experience" [PAS=0] or "brief glimpse" [PAS=1] than "almost clear experience" [PAS=2] or "clear experience" [PAS=3] of the stimulus. (**B) Average percentage of correct responses as a function of the PAS ratings, separated by target location.** The accuracy of the participants increased with awareness.

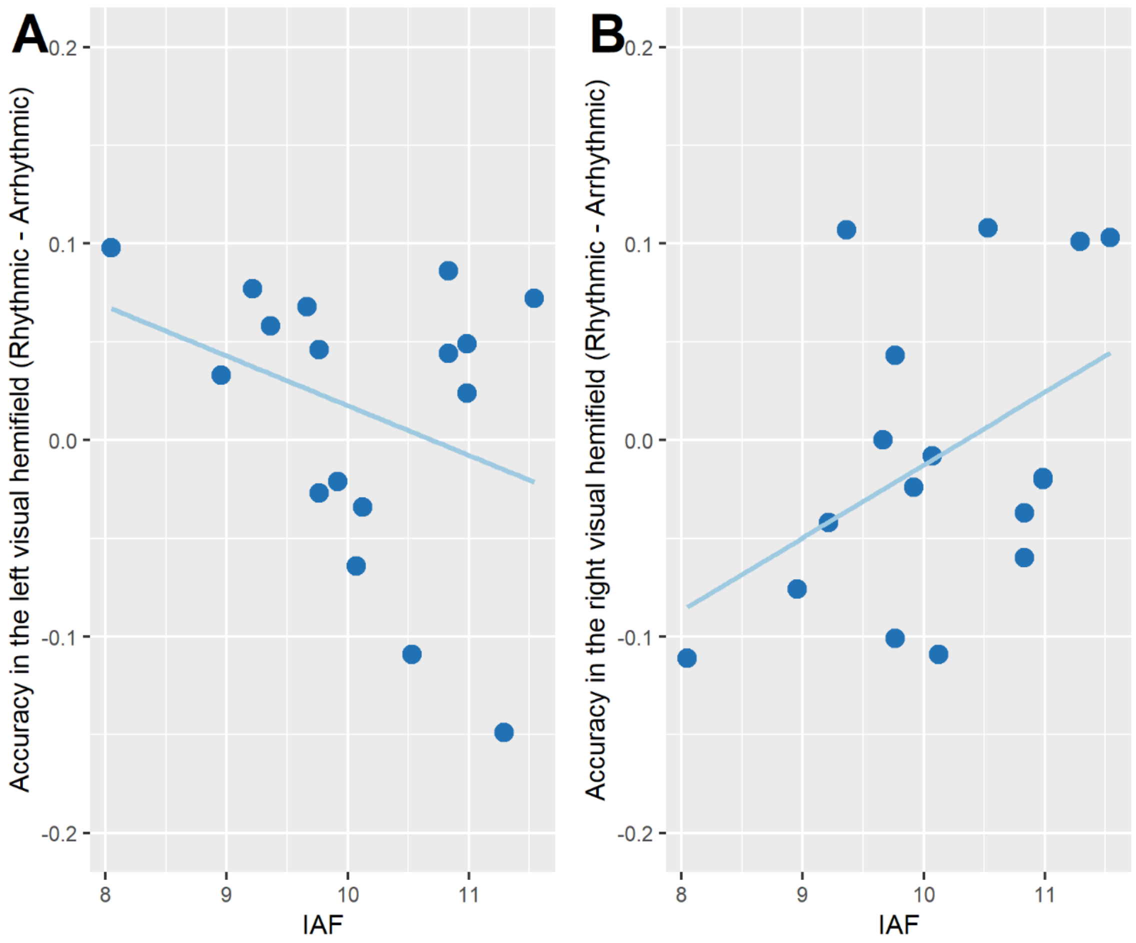

Supplementary Figure 2: Change in accuracy as a function of IAF. (A) Specific effects of entrainment (10Hz-TMS minus ar-TMS) in the left visual hemifield as a function of IAF. **(B)** Specific effects of entrainment (10Hz-TMS minus ar-TMS) in the right visual hemifield as a function of IAF.
